# HHV-6A in vitro infection of thyrocytes and T cells alters the expression of miRNA associated to autoimmune thyroiditis

**DOI:** 10.1186/s12985-016-0672-6

**Published:** 2017-01-11

**Authors:** Elisabetta Caselli, Maria D’Accolti, Irene Soffritti, Maria Chiara Zatelli, Roberta Rossi, Ettore degli Uberti, Dario Di Luca

**Affiliations:** 1 Department of Medical Sciences, Section of Microbiology and Medical Genetics, University of Ferrara, via L. Borsari 46, 44121 Ferrara, Italy; 2 Department of Medical Sciences, Section of Endocrinology and Internal Medicine, University of Ferrara, via A. Moro 8, 44124 Ferrara, Italy; 3 Endocrinology Unit, Azienda Ospedaliero-Universitaria di Ferrara, via A. Moro 8, 44124 Ferrara, Italy

**Keywords:** HHV-6, miRNA, Autoimmune thyroiditis

## Abstract

**Background:**

Human herpesviruses have been hypothesized as environmental triggers in the development of autoimmune thyroid diseases (AITD), and in particular active human herpesvirus 6A (HHV-6A) infection was detected in thyrocytes of Hashimoto’s thyroiditis (HT) patients, who also show specific anti-viral immune responses. On the other hand, AITD patients display modulation of specific miRNAs in thyroid tissue and blood. We wanted to ascertain whether HHV-6A infection might be correlated to the miRNA dysregulation observed in AITD.

**Methods:**

Human thyroid and T-cell lines were infected in vitro with HHV-6A,-6B or −7, and analysed for miRNAs expression, either by microarray or by specific RT-PCR assays detecting miRNAs associated with AITD in vivo.

**Results:**

HHV-6A infection, but not -6B or −7 infections, induced a decrease in miR-155_2 expression and an increase in miR-1238 expression in thyrocytes, as well as an increase in the expression levels of several autoimmunity-associated miRNAs in T lymphocytes, including miR-16_1, miR34a, miR-130a, miR-143_1, miR-202, miR-301b, miR-302c, miR-449b, miR-451_1, and miR-1238_2.

**Conclusions:**

HHV-6A infection modulates miRNAs expression in the cell types involved in the development of AITD. Notably, our in vitro findings correlate with what observed in AITD patients, further supporting the association between HHV-6A infection and AITD development. Moreover, these effects are 6A-specific, emphasizing the differences between the two HHV-6 virus species, and suggesting diverse virus mechanisms of action and therapeutic approaches.

## Background

Autoimmune thyroid diseases (AITD) are very common thyroid disorders, showing increased prevalence in recent years [1]. In addition to genetic background, several viruses, including herpesviruses, have been suggested as possible environmental causes, but conclusive data are still lacking. In particular, we and others reported a high prevalence of active human herpesvirus 6A (HHV-6A) infection in thyrocytes of Hashimoto’s thyroiditis (HT) patients [2, 3], who also display specific cellular and humoral anti-viral responses, increased NK killing of infected thyroid cells [3], and increased CD3-CD56^bright^CD16^−^NK cells, whose activation significantly correlates with plasma levels of anti-thyroid peroxidase (TPO) and anti-thyroglobulin (Tg) autoantibodies [4].

HHV-6 is a ubiquitous virus including two distinct viral species, HHV-6A and HHV-6B, displaying different cellular tropism and receptors usage [5–8]. Although originally considered lymphotropic, the in vivo tropism of both viruses is considerably broader, including macrophages, endothelial cells, salivary glands, and brain [5]. HHV-6B is associated with childhood *exanthema subitum*, whereas HHV-6A is mostly adult-associated, and has been correlated to several chronic autoimmune inflammatory diseases, including AITD [5, 9].

On the other hand, some microRNAs (miRNA) have been found dysregulated in AITD patients and implicated in the disease development. In particular, miR-155_2 has been found decreased and miR-200a1 increased in HT thyroid tissue compared with healthy controls [10], whereas miR-16, miR-22, miR-375 and miR-451 were found increased in serum of HT and Grave’s Disease (GD) patients, compared with controls [11].

Some miRNAs function has been associated with the control of innate and adaptive immune responses [12], but further work is needed to understand the role of miRNAs and their potential as disease markers and/or therapeutic targets in autoimmune thyroiditis. Furthermore, no data are available on the potential ability of HHV-6 to induce miRNAs modulation in cells involved in the disease development (i.e. thyrocytes and lymphocytes).

Based on these observations, the present study was addressed to ascertain whether HHV-6A infection might induce modulation of those miRNAs associated in vivo with AITD, and particularly with HT.

## Methods

### In vitro virus infection

The human thyroid Nthy-ori3-1 and Jurkat T cell lines were used for in vitro infection experiments. The cells were propagated and seeded at optimal density [3], then infected with HHV-6A, −6B or −7 cell-free virus inocula, at a m.o.i. of 10 genome equivalents per cell, as previously detailed [3]. Control cells were infected with the correspondent UV-inactivated viruses, and virus infection was monitored by analyzing virus presence and transcription in infected cells, respectively by real time quantitative PCR (qPCR) and qPCR after retrotranscription (RT-qPCR), amplifying the U42 gene of all viruses, as previously described [3].

### miRNA analysis

The mature miRNA fraction was extracted from infected or control cells by the miRNeasy mini kit (Qiagen, Hilden, Germany), and retro-transcribed as indicated by the manufacturer (miScript RT kit; Qiagen, Hilden, Germany). miRNA fraction was then analysed by a microarray detecting a panel of 84 microRNAs specifically associated to inflammation and autoimmunity (Qiagen, Hilden, Germany), and by specific real time quantitative PCR (qPCR) assays for the following miRNAs: miR-16-1_1, miR-16_2, miR-22, miR-34a, miR-143_1, miR-146a_1, miR-155_1, miR-155_2, miR-181a_1, miR-181a_2, miR-200a_1, miR-200a_2, miR-375, miR-451miR-1238_2. Two assays detecting constitutively expressed cellular miRNAs (miR-RTC and miR-SNORD11) were also used as controls.

### Statistical analysis

Statistical analysis of collected data was performed by Student’s *t* test.

## Results

### Herpesvirus infection in human thyroid and T-cell lines

**Fig. 1:**
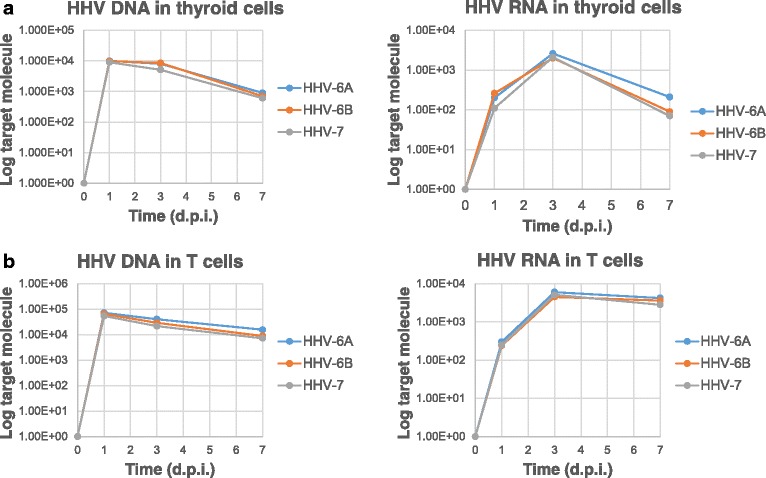
Herpesvirus infection in human thyroid and T lymphoid cell lines. Nthy-ori3-1 (**a**) and Jurkat T cell lines (**b**) were infected with HHV-6A, −6B or −7 cell-free virus inocula (m.o.i. 10:1). Virus presence (DNA) and transcription (RNA) were evaluated respectively by qPCR and RT-qPCR performed on U42 virus genes at 1, 3 and 7 d.p.i., as already detailed [3]

Prior to study miRNA expression, the infection efficiency of the viruses used for the experiments was checked by qPCR in both thyroid Nthy-ori3-1 and lymphoid Jurkat cells, showing that all virus types (HHV-6A, HHV-6B and HHV-7) were equally entering and replicating in both Nthy-ori3-1 and Jurkat T cells (Fig. 1), confirming what previously published [3, 5, 13].

### miRNA expression in infected thyroid and T-cell lines

miRNA expression in infected cells was analysed at 0, 7, 24, 48, 72 h post infection (h.p.i.) and 7 days post infection (d.p.i.). Briefly, the mature miRNA fraction was extracted from infected and control cells, retro-transcribed and analysed by two different qPCR methods. At 48 h.p.i., extracted miRNAs were analysed by a qPCR microarray detecting a panel of 84 microRNAs specifically associated to inflammation and autoimmunity; at all times post infection miRNA fraction was instead analysed by specific qPCR assays detecting the following miRNAs: miR-16-1_1, miR-16_2, miR-22, miR-34a, miR-143_1, miR-146a_1, miR-155_1, miR-155_2, miR-181a_1, miR-181a_2, miR-200a_1, miR-200a_2, miR-375, miR-451miR-1238_2; miR-RTC and miR-SNORD11 were used as internal controls.

**Fig. 2:**
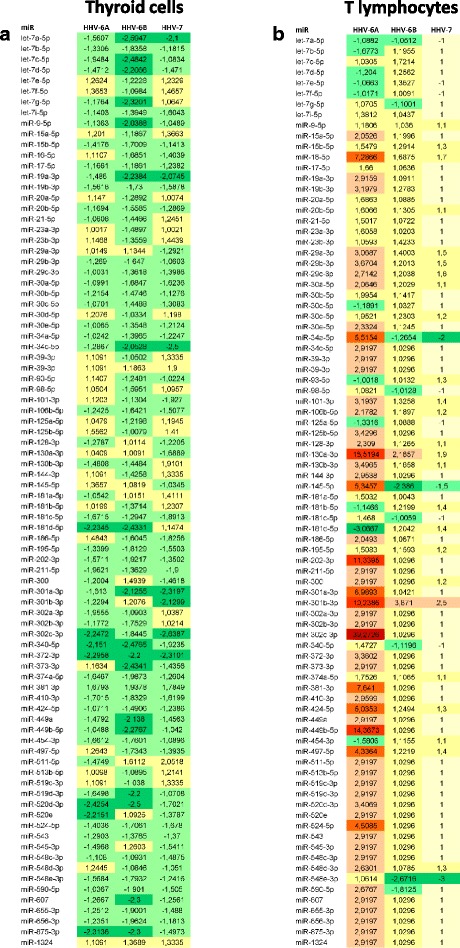
Herpesviruses-induced modulation of autoimmunity-related miRNAs in thyroid and T lymphoid cell lines. Nthy-ori3-1 and Jurkat T cells were uninfected (control) or infected with HHV-6A, HHV-6B or HHV-7 and analysed at 48 h.p.i. for miRNA expression by a microarray analysis evidencing 84 miRNAs associated to inflammation and autoimmunity. Results are expressed as fold-change compared to control values, and represent the mean value of triplicate samples. **a** miRNA expression in thyroid cells **b** miRNA expression in T lymphocytes

As shown in Fig. 2a, in vitro HHV-6A infection of Nthy-ori3-1 thyroid cells did not induce relevant alterations in the expression of over 80 cellular miRNAs associated to inflammation and autoimmunity. In fact, as judged by microarray results at 48 h.p.i., miRNA expression was only modestly modulated (less than 3-fold changes), with most of miRNA resulting slightly downregulated (2.2 fold for miR-181d, miR-302c, miR-372, miR-520e and miR-875; 2.4 fold for miR-520d). Conversely, infection of T lymphoid cells resulted in a very different picture at 48 h.p.i. (Fig. 2b). In fact, HHV-6A infection (but not HHV-6B or HHV-7 infection) induced a remarkable increase in several autoimmunity-related miRNAs, namely: miR-16, miR-34a, miR-130a, miR-202, miR-301b, miR-302c, and miR-449b.

**Fig. 3:**
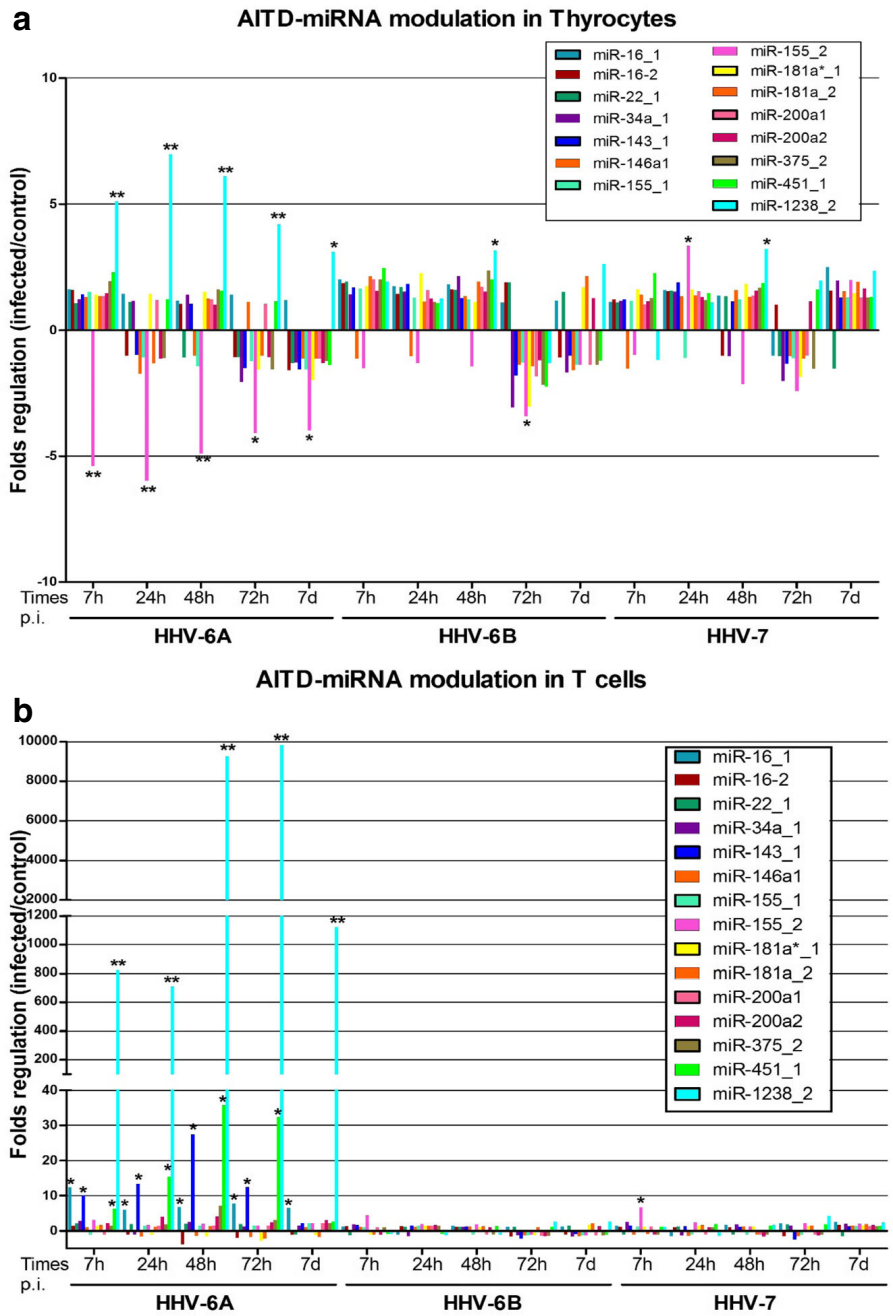
Herpesviruses-induced modulation of AITD-related miRNAs in thyroid and T lymphoid cell lines. Nthy-ori3-1 and Jurkat T cells were uninfected (control) or infected with HHV-6A, HHV-6B or HHV-7 and analysed for expression of individual AITD-associated miRNAs at the indicated times post infection. Results are expressed as fold-change compared to control values, and represent the mean value of triplicate samples in two independent experiments. **a** miRNA expression in thyroid cells (*, *p* < 0.01; **, *p* < 0.001). **b** miRNA expression in T lymphocytes (*, *p* < 0.001; **, *p* < 0.0001)

Contrarily to what observed my microarray analysis, when analyzing individual expression of AITD-associated miRNAs in thyroid cells, results showed that HHV-6A infection, but not HHV-6B or HHV-7 infection, induced in Nthy-ori3-1 thyroid cells an up to 6-fold decrease in miR-155_2 and an up to 7-fold increase in miR-1238_2 levels (Fig. 3a). Virus-induced miRNA modulations were already detectable at 7 h.p.i., persisted till 7 d.p.i., and were statistically significant at all times post infection (*p* < 0.001 and *p* < 0.01).

In parallel, the individual analysis of AITD-related miRNAs in Jurkat T cells evidenced, in HHV-6A infected cells only, statistically significant increases in the expression of miR-16_1 (13-fold peak at 7 h.p.i.), miR-143_1 (28-fold peak at 48 h.p.i.), miR-451_1 (36-fold peak at 48 h.p.i.) and miR-1238_2 (~10^4^-fold increase at 48 and 72 h.p.i.) (*p* < 0.0001 and *p* < 0.001) (Fig. 3b). On the other hand, similarly to what observed in thyroid cells, no significant alteration in miRNA expression were observed in HHV-6B or HHV-7 infected T cells, except for a slight increase in miR-155_2 levels in HHV-7 infected cells at 7 h.p.i.

## Discussion

Our data show for the first time that in vitro HHV-6A infection of human cells of thyroid and T lymphoid origin (namely Nthy-ori3-1 and Jurkat cell lines) induces modulation of miRNAs considered markers of AITD in vivo. Notably, the virus-induced alterations correlate with what observed in vivo, as AITD patients, particularly those affected by HT, have decreased miR-155_2 in thyroid tissue [10], and increased miR-16 in serum [11].

Due to their emerging role as important regulators of immune function [14], the virus-induced dysregulation of these miRNAs might have important consequences in the development of autoimmunity. In particular, miR-155 is a pleiotropic modulator of innate and adaptive immunity [15–17], and might promote autoimmune inflammation [18]. Beside its role as tumor-suppressor [19], miR-16 was recently shown to affect NK function by repressing IFNγ expression [20], and to promote M1 pro-inflammatory type macrophage polarization, affecting T cell activation [21]. No data are instead available on miR-1238 potential functions in immunity, as it was so far recognized only as a tumor-suppressor factor [22]. However, it was identified as a marker of AITD [23], together with miR-143_1, and the strong increase observed in both thyroid and lymphoid HHV-6A infected cells suggest a potential role of miR-1238 in the modulation of immune response, and deserves further studies.

Notably, significant alterations were detected only in HHV-6A infected cells, and not in HHV-6B or HHV-7 infected cells, further strengthening the notion that 6A and 6B species induce diverse effects inside infected cells, and are associated to diverse diseases. Since all used HHV show superimposable structural features and ability to enter and replicate in infected cells, the observed differences might be attributable to factors induced or expressed differently by these viruses inside infected cells. These factors may include both viral and virus-induced cell compounds, which will be interesting to analyze in future studies.

Overall, our current findings add evidences supporting the association between HHV-6A infection and AITD onset, and suggest a potential role of the induced miRNA in the development ofHHV-6A associated diseases. Further studies are however needed to verify these effects in primary human cells and to assess the functional role of virus-induced miRNAs in the development of the virus-induced autoimmunity.
